# Hepatotoxicity Secondary to Levofloxacin Use

**DOI:** 10.7759/cureus.15973

**Published:** 2021-06-27

**Authors:** Ladan Panahi, Salim Surani Surani, George Udeani, Niraj P Patel, Jacob Sellers

**Affiliations:** 1 College of Pharmacy, Texas Agricultural and Mechanical University, Kingsville, USA; 2 College of Medicine, Texas Agricultural and Mechanical University, College Station, USA; 3 Section of Pulmonary - Critical Care & Fellowship Program, Corpus Christi Medical Center, Corpus Christi, USA

**Keywords:** levofloxacin, hepatotoxicity, fluoroquinolones, dili, liver injury

## Abstract

Levofloxacin is a broad-spectrum antibiotic that is used in the treatment of many infections. A rare adverse drug reaction following the use of levofloxacin is drug-induced liver injury. The exact mechanism behind fluoroquinolone-induced liver injury is unknown, but many severe, sometimes fatal hepatotoxicity cases are reported. Current recommendations advise clinicians to discontinue levofloxacin immediately if the patient develops signs and symptoms of hepatitis. This case report presents a 79-year-old male who was prescribed levofloxacin 500 mg by mouth daily for seven days. The patient had a past medical history of dementia, seizures, cerebral vascular accident, pulmonary fibrosis, and chronic kidney disease. Upon admission, the patient began to show signs and symptoms of liver injury. We hereby present a case report and a review of significant literature on levofloxacin-induced liver injury.

## Introduction

Fluoroquinolones are a popular antimicrobial drug class utilized for its broad-spectrum coverage [1], once or twice a day dosing frequency, and high bioavailability allowing for easy intravenous (IV) to per oral (PO) conversion [1]. With the popularity of its use comes an increase of adverse drug reactions such as tendon rupture, prolongation of the Q-T interval, hemolytic uremic syndrome, neuroexcitation, and interstitial nephritis [2,3]. Although many of the adverse drug reactions are well documented in the literature, the specific characteristics of the adverse drug reaction of hepatic injury secondary to levofloxacin use are limited.

Drug-induced liver injury

Drug-induced liver injury (DILI) is a potentially fatal adverse drug event associated with the use of hepatotoxic xenobiotics that causes liver injury, not explained by other causes [4]. DILI is classified as intrinsic or idiosyncratic based on the relationship between the exposure and the resultant liver injury [5]. Medications that cause an intrinsic DILI, such as acetaminophen, produce a predictable dose-dependent liver injury in all individuals [5]. Medications that cause an idiosyncratic DILI produce an unpredictable response in a minority of patients at therapeutic doses [5]. While the mechanism of idiosyncratic DILI is poorly understood, the multiple determinant hypothesis states that it is the result of several factors present simultaneously within the patient related to drug metabolism, transporters, gender, age, concurrent exposures, underlying disease, etc. [5]. DILI can be further classified as hepatocellular injury meaning an injury to the liver cell, cholestatic injury meaning an injury to the biliary system or hepatocytes resulting in intrahepatic cholestasis, or mixed hepatocellular and cholestatic injury that refers to an injury to both [6,7]. Symptoms of DILI vary and include nausea/vomiting, abdominal pain, jaundice, fatigue, and pruritus [6,7]. Symptoms arise after exposure and resolve after discontinuation of the offending agent, except in cases of chronic DILI [6,7]. DILI causes biochemical abnormalities that can present five to 90 days after exposure to the offending agent [8]. In cases of hepatocellular injury, aspartate aminotransferase (AST) and alanine aminotransferase (ALT) will be elevated more than two to five times the upper limit of normal (ULN) [6,7]. In cases of cholestatic injury, alkaline phosphatase (ALP) and conjugated bilirubin will be elevated more than two to five times the ULN [6,7]. In cases of mixed injury, the above biomarkers will be elevated more than two to five times the ULN [6,7]. To differentiate between the three types of DILI, an “R-value” is calculated, where R = (ALT/ULN)/(ALP/ULN). An R-value of ≥5 indicates a hepatocellular injury, an R-value of ≤2 indicates cholestatic injury, and an R-value of >2 to <5 indicates a mixed injury [5]. DILI progresses to chronic injury in 5%-10% of all cases and is associated with approximately 50% of all fulminant hepatic failure cases in the United States [6,7].

Levofloxacin background information

Levofloxacin is a synthetic third-generation fluoroquinolone with spectrum coverage, including aerobic gram-negative and gram-positive organisms as well as atypical organisms [2]. Common indications for levofloxacin use include community-acquired pneumonia, skin and soft tissue infections, urinary tract infections, and sinusitis [2]. Levofloxacin exhibits its bactericidal activity through inhibition of DNA gyrase and topoisomerase IV impeding bacterial synthesis [9]. Levofloxacin is available in IV and PO formulations and is typically dosed once per day [2]. The duration of therapy is typically seven to 14 days depending on the type and severity of indication [2]. The most common adverse effects observed are nausea, headache, diarrhea, insomnia, constipation, and dizziness [9]. Infrequent but serious adverse drug reactions of note include aortic dissection, prolongation of the QT interval, tendon effects as it carries a black box warning for tendon rupture, neurotoxicity that also carries a black box warning, and *Clostridium difficile*-associated diarrhea [2,10]. Metabolism of levofloxacin is limited as approximately 87% is excreted unchanged in the urine [2]. A dose adjustment is necessary in renally impaired patients (creatinine clearance < 50 mL/min) to avoid accumulation as clearance of levofloxacin is substantially reduced and plasma half-life is substantially prolonged. [2] Supplemental doses of levofloxacin are not required in patients on hemodialysis or continuous ambulatory peritoneal dialysis (CAPD) as neither is effective in the removal of the drug from the body [2]. Pharmacokinetic studies in hepatic impairment have not been conducted [2]. The pharmacokinetics of levofloxacin is not expected to be affected by hepatic impairment due to its limited metabolism [2].

## Case presentation

A 79-year-old male was admitted to the hospital unit with the chief complaint of worsening cough and abdominal pain. He was seen in the emergency room three days before the admission with the chief complaint of wheezing and non-productive cough where he was diagnosed with bronchitis and sent home with levofloxacin 500 mg by mouth daily for seven days and albuterol hydrofluoroalkane (HFA) inhaler as needed. The patient had a significant past medical history of dementia, seizures, cerebral vascular accident, pulmonary fibrosis, and chronic kidney disease with a creatinine clearance (CrCL) of 36 mL/min on day 1 of their hospital admission. The patient’s family reports no tobacco and alcohol use. The patient returns to the emergency room due to a lack of symptomatic improvement as well as a new complaint of abdominal pain, fever, and altered mental status per family members. Upon admission, the patient appeared jaundiced with right upper quadrant pain, and all hepatic enzymes were elevated with total bilirubin three times more than the ULN, alkaline phosphatase two times more than the ULN, AST 14 times more than the ULN, and ALT six times more than the ULN (Table 1).

**Table 1:** Pertinent Patient-Specific Laboratory Values During Hospital Admission FQ: Fluoroquinolone; Na: sodium; K: potassium; Cl: chloride, CO_2_: bicarbonate; BUN: blood urea nitrogen; SCr: serum creatinine; CrCL: creatinine clearance; Alb: albumin; T. Bili: total bilirubin; ALP: alkaline phosphatase; AST: aspartate aminotransferase; ALT: alanine aminotransferase; INR: international normalized ratio; PT: prothrombin time.

Day of Readmission	1	2	3	4	5	6	7	8	9
Days after initiation of FQ	4	5	6	7	8	9	10	11	12
Na (135-145 mEq/mL)	131	136	134	134	135	132	135	133	133
K (3.5-5 mEq/L)	4.3	3.5	4.9	4.3	3.5	4.0	-	3.8	3.3
Cl (96-106 mEq/L)	98	103	104	103	103	105	111	104	103
CO_2_ (24-30 mEq/L)	26	22	23	23	25	22	20	24	25
Glucose (mg/dL)	173	459	303	265	172	341	85	331	209
BUN (8-20 mg/dL)	38	40	28	28	29	29	16	19	17
SCr (0.5-1.1 mg/dL)	1.7	1.6	1.3	1.3	1.3	1.2	0.9	1.1	1.1
CrCL (mL/min)	36	39	48	48	48	52	69	56	56
Alb (3.5-5.5 mg/dL)	3.2	3	3	2.9	2.9	2.7	2	2.4	2.5
T. Bili (0.30-1 mg/dL)	3.2	3.2	4.7	4.7	4.8	4.8	4	4	2.4
ALP (30-120 units/L)	268	256	317	295	289	324	296	409	443
AST (<35 units/L)	507	396	318	331	316	205	193	146	92
ALT (<35 units/L)	234	212	252	268	278	242	194	193	158
INR (0.9-1.1)		1.32				1.16			
PT (11.1-13.1 s)		32.7				12.9			

An ultrasound of the abdomen was performed and showed a distended gallbladder with no gallstones and a common bile duct diameter of 5 mm. Upon admission, the patient presented with acute chronic kidney injury, with slightly elevated blood urea nitrogen (BUN), and mild tachycardia of 106 beats per minute (all other vitals normal). Of note, medications before admission that may have potentially led to the abnormalities in the hepatic laboratory work are levofloxacin and rosuvastatin (Table 2). The patient was diagnosed with drug-induced cholestatic hepatitis due to levofloxacin after performing a hepatobiliary iminodiacetic acid (HIDA) scan and autoimmune hepatitis panel to rule out other causes. All hepatic enzymes peaked between day 8 and day 9 of hospital stay and improved thereafter in the remainder of the hospital admission. The patient’s renal function also improved throughout the hospital stay. The patient was discharged home on day 9 with instructions for outpatient follow-up.

**Table 2:** Medications Administered Before Hospital Admission PO: Per oral; BID: twice a day.

Medications Prior to Admission
Clopidogrel 75 mg PO daily
Aspirin 325 mg PO daily
Rosuvastatin 20 mg PO daily
Metoprolol tartrate 12.5 mg PO BID
Novolog 70/30 28 units subQ BID
Levetiracetam 250 mg PO BID
Levofloxacin 500 mg PO daily

## Discussion

Levofloxacin-induced hepatotoxicity

Minor elevations in serum alanine aminotransferase (ALT) and aspartate transaminase (AST) have been observed in 2%-5% of patients in short-term studies of levofloxacin use [11]. The abnormalities were typically asymptomatic, self-limiting, and transient with no intervention necessary [11]. Hepatotoxicity is deemed to be a drug class effect as other fluoroquinolones are associated with this toxicity in published case reports and present abruptly, typically one to three weeks after drug initiation [11]. If patients do present with hepatotoxicity and are symptomatic, the presentation will predominately be a hepatocellular or mixed pattern of injury or jaundice and can potentially lead to hepatic failure or cholestatic hepatitis [11]. Autoantibody synthesis is rare, but immunological presentations such as fever, rash, and eosinophilia can commonly be observed [11]. Although the majority of cases of levofloxacin-induced DILI are self-limiting, some cases of acute liver failure, prolonged jaundice, cholestasis, and vanishing bile duct syndrome have been linked to fluoroquinolones [11]. The pathophysiology of fluoroquinolone-induced hepatotoxicity is unknown. The proposed mechanism relates to hypersensitivity even though the allergy-driven presentation was not present in all cases [11]. Discontinuing levofloxacin leads to complete recovery and resolution of clinical signs and symptoms by four to eight weeks in milder cases [11]. The mainstay of treatment of DILI secondary to levofloxacin is the discontinuation of the medication and not re-challenging the medication as hypersensitivity and liver injury may present again with its use. A cross-sensitivity has been observed among the fluoroquinolones; therefore, avoiding fluoroquinolone use or frequent monitoring for the clinical presentation of DILI is warranted [11].

Literature summary

A comparison of detailed case reports with this case shows some key similarities and differences (Table 3) [12-20]. Case reports in the literature regarding levofloxacin-induced hepatotoxicity are predominantly of males who are above 60-years old with no pre-existing liver dysfunction. One observation that can be made is the impact of levofloxacin on hepatic lab values. Lab value abnormalities were reported commonly in two to seven days after initiation of levofloxacin and peaked typically at a minimum of five days after initiation of the medication. ALT and AST increased over 10 times the ULN in the majority of cases, and elevations in total bilirubin and alkaline phosphatase were also observed in the majority of cases. Our case study demonstrates a similar pattern of lab value abnormalities compared to other cases. Another observation that can be made is the symptoms of levofloxacin-induced hepatotoxicity that the patient experiences. Symptoms varied widely from asymptomatic to abdominal pain/tenderness, jaundice, and altered mental status. The majority of cases demonstrated a recovery time of one to three weeks after discontinuation of the fluoroquinolone. The remaining case studies did not uniformly discuss other factors that may have contributed to the drug-induced liver injuries such as renal function, pre-existing hepatic dysfunction, concurrent medication, and co-morbidities. One contributing factor to the DILI observed in our case study was that the levofloxacin dose was not renally adjusted based on the patient's CrCL at admission and therefore may have played a role in increasing the risk for drug toxicity and adverse drug events associated with levofloxacin use.

**Table 3:** Comparison and Summary of Case Reports in the Literature ALP: Alkaline phosphatase; AST: aspartate aminotransferase; ALT: alanine aminotransferase; WNL: within normal limits; RUQ: right upper quadrant; -: information not reported; BID: twice a day.

Study Name	Levofloxacin Dosing Regimen	Total Daily Dose	Duration of Administration	Onset of Abnormal Lab Value (Days)	Peak ALT (<35 IU/L)	Peak AST (<35 IU/L)	Peak Bilirubin (0.3-1 mg/dL)	Peak ALP (30-120 IU/L)	Symptom Onset (Day)	Clinical Presentation	Resolution of Symptoms and/or Labs (Days)
Coban et al. 2005 [12]	500 mg daily	500 mg	10 days	Day 2	937	1750	32.3	147	Day 2	Yellow sclerae, dark urine, severe fatigue, and malaise	No; death
Levine et al. 2014 [13]	500 mg PO and IV daily	500 mg	74 days	-	614	273	23.6	483	Day 53	Dark urine, jaundice, and hair loss	-
Spahr et al. 2001 [14]	500 mg daily	500 mg	10 days	-	4440	5000	3.67	157	-	Jaundice, dark urine, abdominal discomfort, nausea and vomiting, dehydration	No; death
Karim et al. 2001 [15]	500 mg daily	500 mg	-	Day 2	7071 (Day 5)	4962 (Day 5)	2.5 (Day 7)	90 (Day 16)		None	Yes; 1 week
Carrascosa et al. 2009 [16]	500 mg BID	1000 mg	5 days	-	1577 (Day 10)	1754 (Day 10)	4 (Day 10)	210 (Day 3)	Day 10	Asthenia, jaundice, and dark urine	No; death
Titos-Arcos et al. 2011 [17]	500 mg BID	1000 mg	-	Day 5	52 (Day 7)	58 (Day 5)	WNL	1445 (Day 7)	-	-	Yes; 24 days
Figueira-Coehlo et al. 2010 [18]	500 PO QD	500 mg	7 days	Day 7	953 (Day 13)	329 (Day 13)	1.6 (Day 2)	141 (Day 28)	-	-	Yes; 2 weeks after 28 days of stay
Schwalm and Lee 2003 [19]	250 mg PO Q24H	250 mg	21 days	-	857 (Day 21)	1392 (Day 21)	4.09 (Day 21)	423 (Day 21)	Day 21	Confusion, drowsiness, RUQ tenderness	Yes; 1 week
Liccata et al. 2012 Case 1 [20]	500 mg PO QD	500 mg	5 days	Day 5	400	370	0.6	-	Day 5	Nausea and hyperchromic urine, hepatomegaly	Yes; 2 weeks
Liccata et al. 2012 Case 2 [20]	IV (unknown dose)	-	-	Day 2	1129	587	0.45	225	-	-	Yes; 1 month
Our Patient Case	Levofloxacin 500 mg PO daily	500 mg	7 days	Day 3	278 (Day 5)	507 (Day 1)	4.8 (Day 5-6)	443 (Day 9)	3 days	Abdominal pain, fevers, and altered mental status	-

## Conclusions

The use of levofloxacin was the most probable cause of drug-induced liver injury in this patient. Levofloxacin is not a first-line treatment option in most cases due to its safety profile, and clinicians should be cognizant of the possible hepatotoxic effects associated with fluoroquinolones. Patients at risk for liver injury or dysfunction should avoid the use of levofloxacin and avoid any other antibiotics in the same class due to potential cross-sensitivity. Patients should be educated on documenting DILI as a drug allergy as a means to trigger healthcare providers to avoid prescribing fluoroquinolones in the future. If liver function tests begin to rise rapidly in a patient receiving a fluoroquinolone, discontinuing and initiating another antibiotic are suggested as a precautionary measure. Further studies regarding levofloxacin use associated with liver injury may be warranted to establish stronger recommendations regarding its use.

## References

[1] Orman ES, Conjeevaram HS, Vuppalanchi R, Freston JW, Rochon J, Kleiner DE, Hayashi PH (2011). Clinical and histopathologic features of fluoroquinolone-induced liver injury. Clin Gastroenterol Hepatol.

[2] (2021). LEVOFLOXACIN - levofloxacin injection, solution. http://labeling.pfizer.com/ShowLabeling.aspx?id=4996.

[3] Radovanovic M, Dushenkovska T, Cvorovic I (2018). Idiosyncratic drug-induced liver injury due to ciprofloxacin: a report of two cases and review of the literature. Am J Case Rep.

[4] Suk KT, Kim DJ (2012). Drug-induced liver injury: present and future. Clin Mol Hepatol.

[5] Roth RA JH, Luyendyk JP (2019). Casarett & Doull’s Toxicology: The Basic Science of Poisons.

[6] Baloch ZQ, Raza MA, Abbas SA, Bukhari S (2017). Ciprofloxacin-induced hepatotoxicity in a healthy young adult. Cureus.

[7] Andrade RJ, Lucena MI, Kaplowitz N (2006). Outcome of acute idiosyncratic drug-induced liver injury: long-term follow-up in a hepatotoxicity registry. Hepatology.

[8] Hussaini SH, Farrington EA (2007). Idiosyncratic drug-induced liver injury: an overview. Expert Opin Drug Saf.

[9] Podder V, Sadiq NM (2019). Levofloxacin. https://www.ncbi.nlm.nih.gov/books/NBK545180/.

[10] Tanne JH (2008). FDA adds "black box" warning label to fluoroquinolone antibiotics. BMJ.

[11] Bethesda (MD): National Institute of Diabetes and Digestive and Kidney Diseases (2020). LiverTox: Clinical and Research Information on Drug-Induced Liver Injury [Internet]. LiverTox: Clinical and Research Information on Drug-Induced Liver Injury [Internet].

[12] Coban S, Ceydilek B, Ekiz F, Erden E, Soykan I (2005). Levofloxacin-induced acute fulminant hepatic failure in a patient with chronic hepatitis B infection. Ann Pharmacother.

[13] Levine C, Trivedi A, Thung SN, Perumalswami PV (2014). Severe ductopenia and cholestasis from levofloxacin drug-induced liver injury: a case report and review. Semin Liver Dis.

[14] Spahr L, Rubbia-Brandt L, Marinescu O, Armenian B, Hadengue A (2001). Acute fatal hepatitis related to levofloxacin. J Hepatol.

[15] Karim A, Ahmed S, Rossoff LJ, Siddiqui RK, Steinberg HN (2001). Possible levofloxacin-induced acute hepatocellular injury in a patient with chronic obstructive lung disease. Clin Infect Dis.

[16] Carrascosa MF, Lucena MI, Andrade RJ (2009). Fatal acute hepatitis after sequential treatment with levofloxacin, doxycycline, and naproxen in a patient presenting with acute Mycoplasma pneumoniae infection. Clin Ther.

[17] Titos-Arcos JC, Hallal H, Robles M, Andrade RJ (2011). [Acute cholestatic hepatitis associated with levofloxacin]. Gastroenterol Hepatol.

[18] Figueira-Coelho J, Pereira O, Picado B, Mendonça P, Neves-Costa J, Neta J (2010). Acute hepatitis associated with the use of levofloxacin. Clin Ther.

[19] Schwalm JD, Lee CH (2003). Acute hepatitis associated with oral levofloxacin therapy in a hemodialysis patient. CMAJ.

[20] Licata A, Randazzo C, Morreale I, Butera G, D'Alessandro N, Craxì A (2012). Fluoroquinolone-induced liver injury: three new cases and a review of the literature. Eur J Clin Pharmacol.

